# Spin State Disproportionation in Insulating Ferromagnetic LaCoO_3_ Epitaxial Thin Films

**DOI:** 10.1002/advs.202303630

**Published:** 2023-07-23

**Authors:** Shanquan Chen, Jhong‐Yi Chang, Qinghua Zhang, Qiuyue Li, Ting Lin, Fanqi Meng, Haoliang Huang, Yangyang Si, Shengwei Zeng, Xinmao Yin, My Ngoc Duong, Yalin Lu, Lang Chen, Er‐Jia Guo, Hanghui Chen, Chun‐Fu Chang, Chang‐Yang Kuo, Zuhuang Chen

**Affiliations:** ^1^ School of Materials Science and Engineering Harbin Institute of Technology Shenzhen 518055 China; ^2^ Department of Electrophysics National Yang Ming Chiao Tung University Hsinchu 30010 Taiwan; ^3^ Beijing National Laboratory for Condensed Matter Physics and Institute of Physics Chinese Academy of Sciences Beijing 100190 China; ^4^ Department of Electronic Science East China Normal University Shanghai 200241 China; ^5^ NYU‐ECNU Institute of Physics NYU Shanghai Shanghai 200124 China; ^6^ Hefei National Research Center for Physical Sciences at the Microscale and Anhui Laboratory of Advanced Photon Science and Technology University of Science and Technology of China Hefei 230026 China; ^7^ Department of Physics Faculty of Science National University of Singapore Singapore 117551 Singapore; ^8^ Shanghai Key Laboratory of High Temperature Superconductors Physics Department Shanghai University Shanghai 200444 China; ^9^ Department of Physics Southern University of Science and Technology Shenzhen 518055 China; ^10^ Department of Physics New York University New York NY 10012 USA; ^11^ Max‐Planck Institute for Chemical Physics of Solids Nöthnitzer Str. 40 01187 Dresden Germany; ^12^ National Synchrotron Radiation Research Center 101 Hsin‐Ann Road Hsinchu 30076 Taiwan; ^13^ Flexible Printed Electronics Technology Center Harbin Institute of Technology Shenzhen 518055 China

**Keywords:** epitaxial strain, insulating ferromagnetism, lacoo_3_ thin films, spin state disproportionation, X‐ray absorption spectroscopy

## Abstract

The origin of insulating ferromagnetism in epitaxial LaCoO_3_ films under tensile strain remains elusive despite extensive research efforts are devoted. Surprisingly, the spin state of its Co ions, the main parameter of its ferromagnetism, is still to be determined. Here, the spin state in epitaxial LaCoO_3_ thin films is systematically investigated to clarify the mechanism of strain‐induced ferromagnetism using element‐specific X‐ray absorption spectroscopy and dichroism. Combining with the configuration interaction cluster calculations, it is unambiguously demonstrated that Co^3+^ in LaCoO_3_ films under compressive strain (on LaAlO_3_ substrate) is practically a low‐spin state, whereas Co^3+^ in LaCoO_3_ films under tensile strain (on SrTiO_3_ substrate) have mixed high‐spin and low‐spin states with a ratio close to 1:3. From the identification of this spin state ratio, it is inferred that the dark strips observed by high‐resolution scanning transmission electron microscopy indicate the position of Co^3+^ high‐spin state, i.e., an observation of a spin state disproportionation in tensile‐strained LaCoO_3_ films. This consequently explains the nature of ferromagnetism in LaCoO_3_ films. The study highlights the importance of spin state degrees of freedom, along with thin‐film strain engineering, in creating new physical properties that do not exist in bulk materials.

## Graphical abstract

The long‐standing puzzle of the spin state of Co ions and insulating ferromagnetism in LaCoO_3_ films are demonstrated using element‐specific X‐ray absorption spectroscopy and dichroism. This finding highlights the importance of spin state degrees of freedom, along with thin‐film strain engineering in creating new physical properties and holds great promise for the development of quantum spintronic nanodevices.

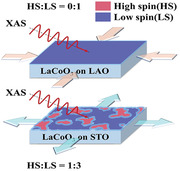

## 1 Introduction

Perovskite oxides exhibit a variety of electronic properties, including high‐temperature superconductivity, colossal magnetoresistance, and multiferroism, as a consequence of complex interplay among lattice, charge, spin, and orbital degrees of freedom.^[^ ^1^, ^2^, ^3^, ^4^, ^5^, ^6^ ^]^ Epitaxial strain can dramatically alter the physical properties of the strongly correlated oxide materials.^[^ ^7^ ^]^ One notable example is the tensile‐strain‐induced ferromagnetism in LaCoO_3_ (LCO) epitaxial thin films.^[^ ^8^, ^9^, ^10^ ^]^ Bulk LCO is a diamagnetic insulator below ≈100 K due to a low‐spin (LS, *t*_2g_^6^ *e*_g_^0^, *S* = 0) ground‐state configuration of Co^3+^, and becomes paramagnetic at higher temperature with the increasing population of Co^3+^ high‐spin (HS, *t*_2g_^4^ *e*_g_^2^, *S* = 2) state.^[^ ^11^ ^]^ Although this intriguing system has been studied extensively for decades,^[^ ^9^, ^12^, ^13^, ^14^, ^15^, ^16^, ^17^ ^]^ the origin of insulating ferromagnetism in tensile‐strained LCO films still remains controversial. Previous investigations have proposed several competing models for the mechanism governing the ferromagnetism in the LCO films,^[^ ^12^, ^13^, ^14^, ^15^, ^18^, ^19^, ^20^, ^21^, ^22^, ^23^, ^24^ ^]^ including orbital order composed of Co^3+^ intermediate spin (IS, *t*_2g_^5^ *e*_g_^1^, *S* = 1) and/or Co^3+^ HS state,^[^ ^13^, ^18^ ^]^ superexchange‐like interaction between LS Co^3+^ and HS Co^2+^ induced by oxygen vacancies,^[^ ^14^, ^15^ ^]^ spin‐state order among Co^3+^ LS and HS^[^ ^12^, ^19^ ^]^ or IS state,^[^ ^20^ ^]^ charge order/disproportionation,^[^ ^21^, ^22^ ^]^ intrinsic twin domains with HS/IS and LS state order, etc.^[^ ^22^, ^23^ ^]^ Apparently, the spin state of Co ions in strained LCO films is a key factor for its ferromagnetism. Yet, its spin states are controversially reported.

Here, to understand the nature of insulating ferromagnetism in epitaxial LCO films, we have engaged a systematic study using soft X‐ray absorption spectroscopy (XAS), X‐ray linear dichroism (XLD) spectroscopy, magnetic circular dichroism (XMCD) spectroscopy and high‐resolution scanning transmission electron microscopy (STEM). Combining with the configuration cluster‐interaction (CI) calculations, we reveal that the Co^3+^ ions in films under tensile strain (on SrTiO_3_, STO) consist of both the LS and HS states and, importantly the ratio of HS to LS in cobalt ions is ≈1:3, whereas the Co^3+^ ions in films under compressive strain (on LaAlO_3_, LAO) possess mainly an LS state. This HS to LS ratio of 1:3 naturally explains the low net magnetic moment of 1 *µ*_B_/Co in the tensile strained films. Microscopically, we investigated the origin of the commonly observed stripe patterns in STEM images with different electron dose conditions. With a high electron dose, we observed a dark‐ to bright‐strip ratio of ≈1:3 in the tensile strained LCO/STO films, whereas with a low electron dose a homogeneously bright image was observed. The images from the LCO/LAO films are electron dose‐independent and have no dark stripes. This indicates that the dark stripes could be the area of HS state, but the formation of the dark stripes due to oxygen vacancies ordering is extrinsic. Important is that there is an intrinsic HS‐ and LS‐state disproportionation in the LCO/STO films. This Co^3+^ HS and LS disproportionation provides a particular ferromagnetic coupling of the Co^3+^ HS intermediated by the Co^3+^ LS for the ferromagnetism in the tensile strained films.

## 2 Results and Discussion

**Figure 1:**
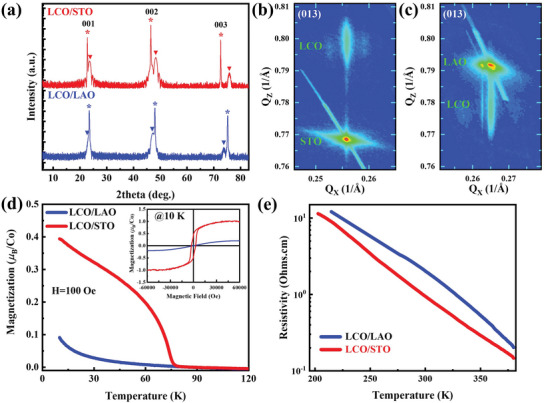
Structural, magnetic, and electrical transport properties of LCO films grown on STO and LAO substrates. a) X‐ray diffraction 2*θ*‐*ω* scan around the 001 and 002 reflections of LCO films grown on STO and LAO substrates. The peaks of substrates and LCO films are indicated with asterisk “*” and inverted triangle “▼”, respectively. b,c) Reciprocal space mappings (RSM) of LCO films grown on STO and LAO around the substrate's (013) reflection, respectively. d) Magnetization–temperature (*M–T*) curves and magnetic hysteresis (*M–H*) loops measured at 10 K (the inset) of LCO films grown on LaAlO_3_(001) and SrTiO_3_(001) substrates. The *M–T* curves were measured at the magnetic field of 100 Oe applied along the film plane. e) Temperature‐dependent resistivity measured under zero magnetic field for 12 nm LaCoO_3_ films grown on LaAlO_3_(001) and SrTiO_3_(001) substrate, respectively.

High‐quality LCO films with a thickness of 12 nm were grown on [001]‐oriented STO and LAO substrates by pulsed laser deposition (see details in Experimental Section). All LCO films have smooth surfaces and are coherently strained to the underlying substrates (**Figure** **1**; Figure S2, Supporting Information). Only (00*l*) diffraction peaks from the films and substrates are visible, indicating that the LCO films are free of impurities (Figure 1a). XRD phi scans indicate the cube‐on‐cube epitaxy growth of the films on the substrates (Figure S3, Supporting Information). Combined with the results of the X‐ray reciprocal‐space mappings (RSM) along the pseudocubic (013) direction (Figure 1b,c), the out‐of‐plane lattice parameter of the LCO films shrink (expand) when the films are epitaxially grown on tensile‐STO (compressive‐LAO) substrates because of the Poisson effect. Furthermore, the Phi‐ angles‐dependent RSMs and rocking curves show the existence of satellite peaks (Figures S4 and S5, Supporting Information), indicating a periodic in‐plane nanodomain modulation (Figure S6, Supporting Information). Figure 1d displays the SQUID magnetometry results for the films. Similar to bulk LCO, the film grown on LAO does not show any discernible ferromagnetic transition or hysteresis loop (Figure 1d). In contrast, LCO grown on STO shows a distinct magnetic transition at 80 K and a clear ferromagnetic hysteresis loop with saturation magnetization of ≈1 *µ*_B_/Co at 10 K, indicates a ferromagnetic order with *T*_C_ ≈80 K. Furthermore, the transport measurement reveals that the LaCoO_3_ films show insulating behavior (Figure 1e). The macroscopic magnetic and transport measurement results are consistent with previous studies.^[^ ^9^, ^15^, ^22^, ^25^, ^26^, ^27^ ^]^

**Figure 2:**
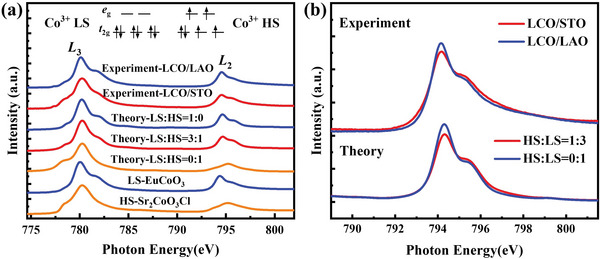
a) Experimental Co *L*_2,3_ XAS spectra taken from LCO films grown on LAO and STO substrates at 30 K, together with the theoretical XAS spectra in the LS‐HS scenario and reference spectra for LS Co^3+^ (EuCoO_3_) and HS Co^3+^ (Sr_2_CoO_3_Cl). b) The experimental Co *L*_2_ XAS spectra of LCO/LAO and LCO/STO at 30 K and the corresponding theoretical isotropic spectra in the LS‐HS scenario.

To figure out the spin states in LCO films, we measured the XAS at Co *L*_2,3_ edges. The XAS at the *L* edges measure the excitations of electrons from the 2*p* core levels to the 3*d* unoccupied states. The strong Coulomb interaction between a 2*p* core‐hole and 3*d* electrons narrows the 3*d* bandwidth and enhances the local solid‐state effect. It makes the XAS a suitable tool to investigate the local spin state and orbital occupation.^[^ ^10^, ^28^, ^29^, ^30^ ^]^ **Figure** **2**a shows the experimental Co *L*_2,3_ XAS spectra taken from LCO films grown on STO and LAO substrates at 30 K. Obviously, Co^3+^ is the main valence state of Co ion in both films. Besides, clear spectral differences between the XAS spectrum of LCO/STO and LCO/LAO are observed (Figure 2b). It is important to note that the XAS difference shown in Figure 2 for LCO between the two different strain states is similar to previous results on bulk LCO measured at high and low temperatures, respectively.^[^ ^10^ ^]^ It indicates a spin‐state difference of Co ions for LCO films under compressive strain and tensile strain.

To look for insight into XAS, we simulate the XAS spectra of Co^3+^ ions with different spin states by employing the CI cluster calculations with full atomic multiplet theory. We first calculated the XAS spectra for Co^3+^ ions in pure LS and pure HS states (Figure 2a). One can see that the calculated XAS spectra for pure HS and LS states nicely reproduce the experimental XAS of Sr_2_CoO_3_Cl and EuCoO_3_ (Figure 2a), in which the former compound has Co^3+^ ions in a pure HS state but the latter in a pure LS state.^[^ ^28^, ^29^, ^30^ ^]^ We next did the linear combination of HS and LS XAS spectra to fit the experimental XAS spectra of LCO films. The curves in Figure 2b show the experimental XAS spectra and theory spectra calculated with a pure LS state (blue) and with HS: LS≈1:3 mixed spin states (red). One can see that the theoretical spectra are in good agreement with the experimental ones. Other cases with different ratios of HS/LS states were also included by CI calculations, but the HS:LS = 1:3 calculated by CI best fits the experimental result (Figure S7, Supporting Information). The results indicate that Co^3+^ in LCO/LAO contains mainly an LS state, while Co^3+^ in LCO/STO is a mixed spin state with an HS to LS ratio of 1:3.

**Figure 3:**
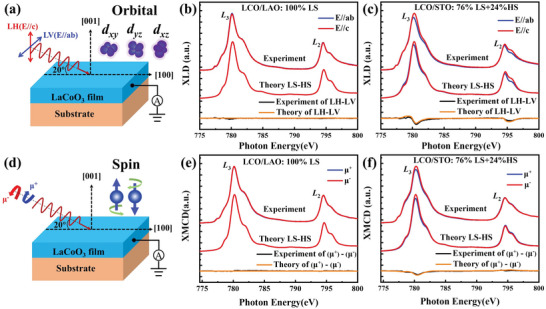
Left panels: A sketch of the a) XLD and d) XMCD measurements. The XLD was obtained from the difference for XAS measured with linear horizontal and linear vertical polarized light. The XMCD was obtained from the difference for XAS measured with left (µ^+^) and right(µ^−^) circularly polarized light. Before the XMCD measurement, the LCO films are first magnetized by a magnetic field of 1 Tesla. In Both XLD and XMCD measurements, the X‐ray beam was incident on the sample at an angle of 20° from the sample surface. Right panels: Top: Experimental Co *L*_2,3_, XAS spectra and XLD at 30 K for b) LCO/LAO and c) LCO/STO, together with the corresponding theoretical XAS and XLD spectra calculated in the LS‐HS scenario. Bottom: Experimental Co *L*_2,3_ XAS spectra and XMCD at 30 K for e) LCO/LAO and f) LCO/STO using circularly polarized X‐rays, together with the theoretical XAS and XMCD spectra for LCO/LAO and LCO/STO calculated in the LS‐HS scenario.

To further confirm the Co^3+^ spin state in the LCO films, we measured the XLD spectra in grazing incidence geometry (**Figure** **3**a). In principle, the XLD originates from two contributions, namely the orbital anisotropy and the magnetic contribution. The XLD spectra shown in Figure 3 were collected after LCO cooling down from room temperature to *T* = 30 K without applying a magnetic field. One can thus expect no long‐range magnetic order presenting in LCO and the contribution from magnetism to XLD is minimal. That is, the orbital anisotropy should be the main origin for causing the XLD signal. Under the LS state, six electrons fully occupy six *t*_2g_ orbitals, making the ground state wavefunction nearly spherical symmetry. There is thus no orbital anisotropy and the XLD is expected to be rather small for Co^3+^‐ion under the LS state. In Figure 3b, one sees that the XLD signal of Co^3+^‐ion in the compressive LCO film is negligibly small, indicating essentially no orbital anisotropy for the LS state. The XAS spectra calculated for *E*//*c* and *E*//*ab* shown in Figure 3b under a pure LS state generate no XLD signal, which reproduces the experimental results and reveals the nearly 100% LS state for Co^3+^‐ion in the compressive LCO/LAO film.

In contrast, as shown in Figure 3c, the tensile‐strained LCO/STO film exhibits clear linear dichroism. For an HS state of Co^3+^‐ion, five majority spin electrons, half‐filling the 3*d* orbitals, make a spherical symmetry of wavefunction, and a minority spin electron occupying a specific orbital can cause an orbital anisotropy and give rise to XLD signals for the film. The XLD signal in Figure 3c clearly shows that the intensity of XAS for *E*//*c* is stronger than that of *E*//*ab*. This indicates that a portion of Co^3+^ cations is an HS state in the tensile‐strained LCO film, and the orbital occupied by the minority spin electron of the Co^3+^ ions is in the *ab*‐plane of the film. To address the HS contribution to XLD, we calculated the XAS spectra of HS Co^3+^ ions by considering the energy of *d*_xy_ orbital lower than that of *d*_xz_/*d*_yz_ by ≈50 meV. A minority spin electron of HS Co^3+^ ions thus occupies the *d*_xy_ orbital and causes the orbital anisotropy, which fully accords to the tensile‐strain status of the LCO/STO film. To simulate the experimental XLD spectrum of LCO/STO, we have carried out a simple simulation by making a superposition of the calculated HS and LS XLD spectra. We found that the spectra with an HS to LS ratio of 1:3 reproduce the experimental spectra the best as shown in Figure 3c, which is consistent with the ratio obtained for simulating the isotropic XAS spectrum. Another piece of important information is that this occupied *d*_xy_ orbital of HS Co^3+^ may explain the in‐plane magnetic easy axis in LCO/STO films.^[^ ^31^ ^]^

The presence of Co^3+^ HS in LCO/STO films offers the essential for its ferromagnetism. To clarify the contribution of Co^3+^ HS state to ferromagnetism, we carried out remanent XMCD experiments on magnetized LCO/LAO and LCO/STO at *T* = 30 K (Figure 3d). Figure 3e,f shows experimental XAS spectra taken with circularly polarized light on the LCO films. The XMCD measurement demonstrates a consistent result with the above macroscopic magnetic properties measured in magnetometry. Spectra of LCO/LAO (Figure 3e) show an almost zero dichroism signal, indicating no spontaneous magnetization. Spectra of LCO/STO (Figure 3f) exhibit a clear dichroism signal, addressing a net ferromagnetic order of the Co^3+^ ions at 30 K. To address the origin of the XMCD, we again simulate the XMCD spectra by CI cluster calculations. We take the same ratio of two spin states found by both the XAS and XLD simulations, a pure LS state for LCO/LAO film and HS:LS = 1:3 for LCO/STO film, to calculate the XMCD spectra. The results are also included in Figure 3. It is not surprising to see that the calculated XMCD for LCO/LAO is zero because of a pure LS (*S* = 0) state. The calculation for LCO/STO with HS:LS = 1:3 exhibits a clear XMCD signal and the calculated spectra nicely reproduce the experimental spectra. Hence, it is the HS Co^3+^‐ions that contribute to the XMCD signal and form the ferromagnetic order. It is worthwhile to mention that this HS to LS ratio of 1:3 naturally explains the low net magnetic moment of ≈1 *µ*_B_/Co in the LCO/STO films from the above SQUID magnetometry results. We also use first‐principles calculations to study the LS state of LCO under LAO substrate and a mixed‐spin state (HS:LS = 1:3) of LCO under STO substrate. In both spin configurations, an insulating state is found in the calculations. In the mixed‐spin state, calculations also find a net magnetization of ≈1 *µ*_B_ per formula (Figure S8, Supporting Information).

**Figure 4:**
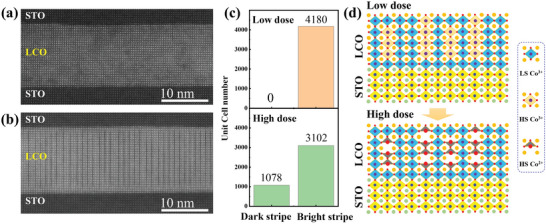
High‐resolution HAADF STEM image of a LCO film grown on an STO substrate with an STO capping layer on top with different electron dose condition; a) low‐ and b) high‐dose conditions. c) Unit cell number containing dark and bright stripe at low and high dose conditions, respectively. d) The schematic diagram of the LaCoO_3_/SrTiO_3_(001) under low electron dose condition and LaCoO_3‐δ_/SrTiO_3_(001) under high electron dose condition. The dotted box on the right side of the schematic denotes the unit cell with LS Co^3+^, HS Co^3+^, and HS Co^2+^, respectively. The yellow substrate represents SrTiO_3_(001).

So far, using high‐resolution STEM lattice modulations in the form of atomic‐scale stripe patterns were commonly observed.^[^ ^14^, ^15^, ^22^, ^23^, ^27^, ^32^, ^33^, ^34^ ^]^ However, not only the origin of the dark stripes observed in LCO/STO has been debated,^[^ ^14^, ^22^, ^23^, ^27^, ^32^, ^33^ ^]^ but also various spin state models were proposed.^[^ ^14^, ^23^, ^32^ ^]^ To clarify the relationship between the microstructure and the Co^3+^ spin state, we performed high‐resolution STEM measurements on LCO/LAO and LCO/STO films (**Figure** **4**; Figure S9, Supporting Information). We paid special attention to the used electron dose, since the STEM technique is not a non‐destructive technique, a high‐energy electron beam could damage/reduce the LCO thin films to a certain extent, resulting in a change in its stoichiometry.^[^ ^35^ ^]^ Figure 4 shows the high‐angle annular dark‐field (HAADF) STEM images of different stages of the beam exposure experiment on LCO/STO. Under low‐dose electron beam irradiation (≈10^6^ e^−^/angstrom^2^), no dark stripes are seen in the STEM image (Figure 4a). However, as the electron beam irradiation dose increases (≈10^8^ e^−^/angstrom^2^), we observed clearly ordered dark stripes in the STEM image (Figure 4b). According to statistics, the ratio of the unit cells where the dark and bright stripes are located is ≈1:3 (Figure 4c). In contrast, no dark stripes were observed in STEM images for compressive LCO film grown on LAO under both low‐dose and high‐dose electron beam irradiation conditions (same irradiation as LCO/STO) (Figure S9, Supporting Information). These results are consistent with previous literature reports.^[^ ^14^, ^15^, ^22^, ^23^ ^]^ Although the exact origin of dark stripes cannot be directly determined by STEM, however, this invites a comparison with the HS–LS ratio of 1:3 found by the above‐mentioned XAS results. It is reasonable to speculate that the position of the dark stripes is not random, but maybe the place where the high‐spin state Co^3+^ is (Figure 4d). We have further verified this assignment by using electron‐energy‐loss spectroscopy (EELS) (Figure S11, Supporting Information). For LCO/LAO, there is no difference in the shape, intensity, and peak position of the O‐*K* and Co‐*L* edges of bright stripes in different regions. However, the clear differences between the EELS in the dark (blue line) and bright stripes (red line) in the LCO/STO film are seen. The front peak (light golden area) intensity of the O‐*K* edge corresponding to the dark stripes is lower than that of the bright stripes pattern, which indicates the formation of oxygen vacancies along these places. In addition, the dark stripes have a higher *L*_3_/*L*_2_ peak intensity ratio than the bright stripes at the Co‐*L* edges. These findings indicate that the oxidation state of Co along the dark stripes has been reduced by a relatively high dose of electron beam. It is well‐known that the metal ion‐ligand bond of HS is weaker than that of LS, therefore, the oxygen ions around the HS are likely to be knocked out more easily.^[^ ^36^, ^37^, ^38^, ^39^ ^]^ This HS‐dark stripe and LS‐bright stripe assignment are also supported by the bond‐length modulation observation by STEM studies as the ionic radius of HS Co^3+^ is larger than that of LS Co^3+^.^[^ ^32^ ^]^ We conclude that the commonly observed dark stripes in LCO/STO films are an extrinsic reduction of oxygen by a high‐dose electron beam used in STEM. Yet, most importantly, it gives access of a direct atomic‐scale positioning of HS Co^3+^ ions, evidencing an intrinsic HS and LS disproportionation in LCO/STO films.

We in fact restore the HS and LS disproportionated insulating phase for bulk LCO at intermediate temperature.^[^ ^40^ ^]^ The advance of LCO/STO film is that by strain engineering, this HS and LS disproportionation is realized at low temperature or as the ground state. The spin state of the Co^3+^ ions in LCO is mainly determined by the competition between the crystal‐field splitting Δ_CF_ and the Hund's exchange interaction *J*_H_.^[^ ^10^ ^]^ The crystal field splitting results from the Co‐ligand interaction, which splits the five degenerate 3*d* orbitals of the Co‐ion into two groups, *e*_g_ and *t*_2g_, with the energy of *t*_2g_ lower than that of *e*_g_. The magnitude of the crystal field splitting is determined by the Co─O bond length. The smaller the Co─O bond length, the larger the crystal field splitting. Therefore, the crystal field splitting favors six electrons of Co^3+^ to fill the *t*_2g_ orbital, forming the LS state. However, the LS state violates Hund's rule, which makes it unstable with respect to the Coulomb repulsion energy. The Co─O bond length, extended by any disturbances such as thermal expansion, would suppress the crystal field splitting and induce the HS state to stabilize the Coulomb repulsion energy. Therefore, the pure LS spin state of LCO is observed only at low temperatures.^[^ ^10^ ^]^ The LCO film in this work under tensile strain has an average Co─O bond length longer than that of one under compressive strain.^[^ ^41^ ^]^ Apparently, the tensile strain enforced by STO is on the border of LS and HS formation.^[^ ^19^ ^]^ The longer Co─O bond length would weaken the crystal field splitting, causing the system to select some region in the film to stabilize in a state following Hund's rule to form the HS state. This explains the appearance of the HS state in the tensile LCO film.

**Figure 5:**
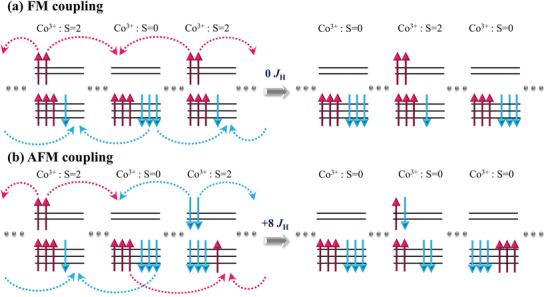
The schematic diagram of the magnetic interactions between HS and LS Co^3+^ ions with a) ferromagnetic and b) antiferromagnetic coupling. Its virtual hopping processes are as follows. The two *e*_g_ electrons of an HS Co^3+^ site hop evenly left and right to the neighboring LS Co^3+^ sites. Two of the six *t*_2g_ electrons of each LS Co^3+^ site do a similar hopping but among *t*_2g_ orbitals. This virtual hopping process prefers a ferromagnetic coupling because an antiferromagnetic coupling will lead to a wrong spin state configuration with an additional energy cost of 8*J*_H_ with respect to a ferromagnetic coupling. The blue dots represent LS Co^3+^ sites. The number of the LS Co^3+^ intermediate between the HS Co^3+^ sites can be altered.

The HS and LS state disproportion in LCO/STO plays an important role in its magnetism.^[^ ^12^, ^19^ ^]^ **Figure** **5** displays a sketch of the superexchange‐like process of t_2g_ and *e*_g_ electrons in an HS–LS‐HS configuration for both ferromagnetic and antiferromagnetic couplings. In the ferromagnetic coupling model, the two *e*_g_ electrons of an HS Co^3+^ site jump equally left and right to the neighboring LS Co^3+^ sites. Two of the six *t*_2g_ electrons of each LS Co^3+^ site perform a similar hopping but between *t*_2g_ orbitals. The mutual jumping of the electrons results in no net charge transfer and ends up in the same spin state configuration as the initial state (Figure 5a). In contrast, the antiferromagnetic coupling brings the system to a different spin state configuration (Figure 5b). It is clear to see that the ferromagnetic coupling is energetically more favorable than the antiferromagnetic coupling by 8*J*_H_. Thus, the system prefers the ferromagnetic coupling as the ground state. We note that this energetic superiority of the ferromagnetic coupling of Co^3+^ ions also holds for configurations such as HS–LS–LS–HS and HS–LS–LS–LS–HS and so on, once the Co^3+^ HS is mediated by the LS. The event of each electron jumps costs the energy of the Coulomb repulsion. Thus, the system remains as an insulating state under ferromagnetic coupling. The insulating ground state of tensile strain LCO film was further supported by our DFT calculations (Figure S8, Supporting Information).

## 3 Conclusion

In summary, using a combination of element‐specific X‐ray absorption spectroscopy and dichroism together with the configuration interaction cluster calculations, we unambiguously demonstrate that Co ions in LCO/LAO thin films under compressive strain are practically a low‐spin state, whereas Co ions in LCO/STO thin films under tensile strain have mixed high‐spin and low‐spin states with a ratio close to 1:3. From the identification of this spin state ratio, we infer that the dark strips in LCO/STO observed by STEM indicate the position of Co^3+^ high‐spin state. Yet, these dark strips are extrinsically caused by the high dose of electron beam used in STEM. Most importantly, this provides access to experimentally observe the Co^3+^ HS and Co^3+^ LS state disproportionation in LCO/STO. In such an HS and LS state disproportionation, the ferromagnetic coupling of the Co^3+^ HS intermediated by Co^3+^ LS is energetically more favorable than the antiferromagnetic coupling.

## 4 Experimental Section

### LaCoO_3_(LCO) Films Growth and Structural Characterization

High quality LCO films (≈12 nm) were grown on [001]‐oriented single crystal SrTiO_3_(STO) and LaAlO_3_(LAO) substrates by pulsed laser deposition (Arrayed Materials RP‐B). During the film deposition, the KrF excimer laser (*λ* = 248 nm) energy density and ablation repetition were given 1.5 J cm^−2^ and 3 Hz, respectively. The deposition temperature was kept at 700 °C, and the oxygen pressures during film deposition was maintained at 20 Pa. All films were cooled down (10°C min^−1^) to room temperature with 10 000 Pa of oxygen atmosphere to ensure the oxygen stoichiometry. High‐resolution X‐ray diffraction (XRD) measurements were performed on a Rigaku‐Smartlab, 9 KW diffractometer with Cu *K*_α1_ radiation. The morphology was determined by an Asylum Research MFP‐3D‐Infinity atomic force microscopy (AFM).

### Electronic Transport and Magnetic Measurements

The magnetic properties of the LCO films were measured using a Quantum Design Superconducting quantum interference device (SQUID) magnetometry measurements. The Magnetization–temperature (*M–T*) curves were measured at the magnetic field of 100 Oe applied along the films plane (in plane) after the field cooling in the same field. The in‐plane magnetization‐magnetic field hysteresis loops (*M–H* curves) were measured at 10 K with magnetic field up to ±6 T. DC transport measurements were carried out in a Quantum Design Physical Property Measurement System (PPMS) from 200 to 380 K under zero magnetic field.

### Soft X‐Ray Spectroscopy Measurements

X‐ray spectroscopy measurements were carried out in the total electron yield mode (TEY) at the TLS11A and TPS45A beamline of the National Synchrotron Radiation Research Center (NSRRC) in Taiwan. The X‐ray absorption spectroscopy (XAS) measurements were performed at 30 K temperature without applying magnetic field using TEY mode and an X‐ray angle of incidence of 20° to the sample surface. X‐ray linear dichroism (XLD) measurements were performed at 30 K and obtained from the difference of horizontal and vertical polarized light absorption spectra without applying magnetic field using TEY mode. The X‐ray beam was incident on the sample at an angle of 20° from the sample surface. X‐ray magnetic circular dichroism (XMCD) spectra were measured in the TEY mode without applying magnetic field using fixed X‐ray circular polarization at 30 K in grazing incidence geometry. Before the XMCD measurement, the LCO films were first magnetized by a magnetic field of 1 Tesla.

### High‐Resolution STEM Characterizations

For cross‐sectional microscopy, a sample was prepared by using focused ion beam (FIB) milling. Cross‐sectional lamellas were thinned down to 60 nm thick at an accelerating voltage of 30 kV with a decreasing current from the maximum 2.5 nA, followed by fine polish at an accelerating voltage of 2 kV with a small current of 40 pA. The atomic scale HAADF‐STEM images of LaCoO_3_ films were performed by Cs‐corrected JEM ARM200CF microscope operated at 200 kV using a high‐angle annular detector for Z‐contrast imaging; and the beam convergence angle was 28.5 mrad and a collection angle of 90–370 mrad.

### Configurational Cluster‐Interaction (CI) Calculations

This theoretical approach considers the fully atomic multiplet effect and configurational interaction as well as local solid effect. Three configurations, namely $|3d^{6}\rangle ,|3d^{7}{\underline{L}}^{1}\rangle$, and$|3d^{8}{\underline{L}}^{2}\rangle$ were employed in the calculations, where $|{\underline{L}}^{n}\rangle$ denotes the number of the holes on the ligand orbitals that make the bond with the Co^3+^ ion. These ligand orbitals were formed by the linear combination of oxygen 2*p* orbitals with respect to the local point symmetry of the Co^3+^ ion. The energy centers of each configuration are defined as |3*d* ^6^〉 = 0, $|3d^{7}{\underline{L}}^{1}\rangle =\Delta$, and $|3d^{8}{\underline{L}}^{2}\rangle =2\Delta +U_{dd}$, where the charge transfer energy Δ  =  2.0 eV and the Coulomb repulsion *U*_dd_ =  5.5 eV were used in the calculations. The hybridization between these three configurations was determined by the parameter of $\sqrt{3}pd\sigma$ that controlled the electron hopping between 3*d*_eg_ and ${\underline{L}}_{\mathrm{eg}}$, and the parameter of 2*pd*π, which controlled the electron hopping between 3*d*_t2g_ and ${\underline{L}}_{t2g}$. *pd*σ  =  1.7*eV* and *pd*π  =  0.74 *eV* were taken for both high‐spin and low‐spin simulations. The electron–electron full‐multiplet effect, which is a key determinant of the Co^3+^ spin state, was included in each configuration. The Slater integrals of F_2_(dd) = 12.662(eV)^*^SIred, F_4_(dd) = 7.916(eV)^*^SIred, which were determined by the Hartree–Fock approximation using the R. D. Cowan code RCN36K, were used in the calculations to account for the atomic full‐multiplet effect, where the parameters for Slater integral reductions (SIred = 0.75) were further employed to compromise the solid‐state effect.

In Figure S10a (Supporting Information) the total energy level diagram is plotted as a function of 10Dq. It can be seen that the ground state of the cluster is either low‐spin (LS) or high‐spin (HS) with a crossover at ≈10Dq ≈0.45 eV. The 10Dq = 0.62 eV and 10Dq = 0.2 eV were chosen to calculate the LS and HS XAS, respectively. It is addressed that the reason of such low 10Dq that could achieve the HS–LS crossover was because of the presence of hybridization between Co^3+^ 3*d* and oxygen ligand orbital, which effectively enhanced the splitting between *e*_g_ and *t*_2g_ level. In Figure S10b (Supporting Information), the total energy level diagram is plotted as a function of 10Dq under the single cluster calculation considering only |3*d* ^6^〉 configuration, that is, the hybridization between Co^3+^ 3*d* and oxygen ligand orbital is completely excluded. It could be seen that the HS–LS transition then increased to 10Dq≈2.0 eV.

To simulate LS and HS XAS, three configurations of $|\underline{c}3d^{7}\rangle ,\;\left|\underline{c}3d^{8}{\underline{L}}^{1}\right\rangle$, and $|\underline{c}3d^{9}{\underline{L}}^{2}\rangle$ were employed, where $\underline{c}$ denotes a hole that appeared in the Co 2*p* core level after an electron transition to the 3*d* orbital by X‐ray. The energy centers of these three configurations are $|\;\underline{c}3d^{7}\rangle =\varepsilon_{\mathrm{XAS}}\;,\;\left|\;\underline{c}3d^{8}{\underline{L}}^{1}\right\rangle =\varepsilon_{\mathrm{XAS}}\;+\Delta +U_{\mathrm{dd}}-U_{\mathrm{dp}}$, and $|\;\underline{c}3d^{9}{\underline{L}}^{2}\rangle =\varepsilon_{\mathrm{XAS}}\;+2\Delta +3U_{\mathrm{dd}}-2U_{\mathrm{dp}}$, where the ε_XAS_ is the energy required to excite an electron from the Co 2*p* to the Co 3*d* level. The *U*_dp_= 7.0 eV was used and the Slater integrals of F_2_(d‐p) = 7.899*SIred, G_1_(d‐p) = 5.947*Sired, and G_3_(d‐p) = 3.384*SIred (with Sired = 0.75) for the Co 2*p*‐3*d* multiplet were further included in the calculation.

### First‐Principle Calculations

First‐principles calculations were performed using the projector augmented wave method,^[^ ^42^ ^]^ as implemented in the Vienna Ab initio Simulation Package (VASP).^[^ ^43^ ^]^ The cutoff energy was 600 eV. The self‐consistent convergence criterion was 10^−6^ eV. The force convergence criterion was 0.01 eV Å^−1^. The pressure convergence threshold on the simulation cell was 1 kbar. The generalized gradient approximation was used with the Perdew–Burke–Ernzerhof parameterization (GGA‐PBE) as the exchange‐correlation functional.^[^ ^44^ ^]^ A 9 × 9 × 6 Monkhorst–Pack grid^[^ ^45^ ^]^ was used as the Brillouin zone integration. To model the correlation effects on Co 3*d* orbitals, the rotationally invariant Hubbard *U* method was used.^[^ ^46^ ^]^ Following the previous study,^[^ ^47^ ^]^ it was found that a *U*_Co_ = 6 eV reproduced the insulating properties of LaCoO_3_ thin films in both low‐spin, high‐spin, and mixed‐spin states. *U*_La_ = 11 eV was turned on for La‐4*f* orbitals so that they were moved away from the Fermi level. Bulk LaCoO_3_ had a crystal structure of space group $R\bar{3}c$. To simulate epitaxial strain, the previous study was followed and tetragonal conventional cell was adopted using the starting ionic positions as mapped from bulk LaCoO_3_. The study defines *a* and *b* as the in‐plane lattice parameters, whereas *c* is the out‐of‐plane lattice parameter. To simulate the epitaxial LaCoO_3_ thin films on different substrates, the in‐plane lattice constant *a* = *b* = 5.57 Å (SrTiO_3_) and *a* = *b* = 5.39 Å (LaAlO_3_) were constrained, respectively. The out‐of‐plane lattice constant *c* and all the internal coordinates were fully relaxed. The conventional cell has four formula units (20 atoms). In the low‐spin (LS) configuration, all the cobalt atoms were low‐spin states. In the mix spin (MS) configuration, one of the cobalt was set to be in the high‐spin state and the other three cobalt atoms were in the low‐spin state.

The study shows the atomic projected density of states (PDOS) in Figure S3 (Supporting Information). Panel (a) is the PDOS of the LaCoO_3_ thin film under a LaAlO_3_ substrate (compressive strain). The calculations were done in a low‐spin state, which yielded a gap of 1.63 eV. The conduction band edge was dominantly Co‐*d* states, while the valence band edge was mainly O‐*p* states that were strongly hybridized with Co‐*d* states. Panel (b) is the PDOS of the LaCoO_3_ thin film under a SrTiO_3_ substrate (tensile strain). Motivated by the experimental results, a mixed‐spin state was calculated in which 75% Co atoms were in a low‐spin state and the 25% Co atoms were in a high‐spin state. It was found that the mixed‐spin state was also insulating with a slightly smaller gap of 1.48 eV. The high‐spin Co‐*d* state (red curve) had a strong splitting between spin‐up and spin‐down channel and yielded a magnetic moment of 3.2μ_B_/Co. The splitting was roughly on the order of *U*_Co_ = 6 eV. The low‐spin Co‐*d* state (green curve) did not generate a net magnetization. The mixed‐state had a net magnetization of 1μ_B_ per formula.

## Conflict of Interest

The authors declare no conflict of interest.

## Supporting information

Supporting Information

## Data Availability

The data that support the findings of this study are available from the corresponding author upon reasonable request.
