# Supplementary material for: Spin State Disproportionation in Insulating Ferromagnetic LaCoO3 Epitaxial Thin Films
Source: Adv Sci (Weinh). 2023 Jul 23;10(27):2303630. doi: 10.1002/advs.202303630 (PMC10520649; doi:10.1002/advs.202303630)
Supplement: Supplementary file 1 — Supporting Information [file ADVS-10-2303630-s001.pdf]

ADVANCED SCIENCE
Open Access

Supporting Information

for *Adv. Sci.*, DOI 10.1002/advs.202303630

Spin State Disproportionation in Insulating Ferromagnetic $LaCoO_3$ Epitaxial Thin Films

Shanquan Chen, Jhong-Yi Chang, Qinghua Zhang, Qiuyue Li, Ting Lin, Fanqi Meng, Haoliang Huang, Yangyang Si, Shengwei Zeng, Xinmao Yin, My Ngoc Duong, Yalin Lu, Lang Chen, Er-Jia Guo, Hanghui Chen, Chun-Fu Chang, Chang-Yang Kuo and Zuhuang Chen**

Supporting Information

Spin State Disproportionation in Insulating Ferromagnetic $LaCoO_3$ Epitaxial Thin Films

Shanquan Chen, Jhong-Yi Chang, Qinghua Zhang, Qiuyue Li, Ting Lin, Fanqi Meng, Haoliang Huang, Yangyang Si, Shengwei Zeng, Xinmao Yin, My Ngoc Duong, Yalin Lu, Lang Chen, Er-Jia Guo, Hanghui Chen, Chun-Fu Chang, Chang-Yang Kuo and Zuhuang Chen**

S. Q. Chen, Y. Y. Si, Prof. Z. H. Chen
School of Materials Science and Engineering, Harbin Institute of Technology, Shenzhen, 518055, China
E-mail: zuhuang@hit.edu.cn
J.-Y. Chang, M.N. Duong, Prof. C.-Y. Kuo
Department of Electrophysics, National Yang Ming Chiao Tung University, Hsinchu, 30010, Taiwan
E-mail: changyangkuo@nycu.edu.tw
Dr. Q.H. Zhang, T. Lin, F.Q. Meng, Dr. E.-J. Guo
Beijing National Laboratory for Condensed Matter Physics and Institute of Physics, Chinese Academy of Sciences, Beijing 100190, China
Q.Y. Li
Department of Electronic Science, East China Normal University, 200241, Shanghai, China
Q.Y. Li, Prof. H. H. Chen
NYU-ECNU Institute of Physics, NYU Shanghai, Shanghai 200122, China
Dr. H.L. Huang, Prof. Y.L. Lu
Hefei National Research Center for Physical Sciences at the Microscale and Anhui Laboratory of Advanced Photon Science and Technology, University of Science and Technology of China, Hefei 230026, China
Dr. S.W. Zeng
Department of Physics, Faculty of Science, National University of Singapore, Singapore 117551, Singapore
Prof. X.M. Yin
Shanghai Key Laboratory of High Temperature Superconductors, Physics Department, Shanghai University, Shanghai 200444, China
Prof. L. Chen
Department of Physics, Southern University of Science and Technology, Shenzhen, 518055, China
Prof. H. H. Chen
Department of Physics, New York University, New York, NY, 10012, USA
Dr. C.-F. Chang

Max-Planck Institute for Chemical Physics of Solids, Nöthnitzer Str. 40, 01187 Dresden, Germany
Prof. C.-Y. Kuo
National Synchrotron Radiation Research Center, 101 Hsin-Ann Road, Hsinchu, 30076, Taiwan
Prof. Z. H. Chen
Flexible Printed Electronics Technology Center, Harbin Institute of Technology, Shenzhen, 518055, China

Keywords: $LaCoO_3$ thin films, Spin state disproportionation, Insulating ferromagnetism, Epitaxial strain, X-ray absorption spectroscopy

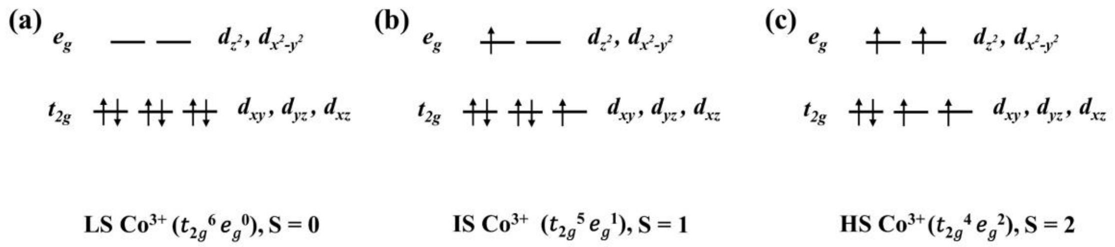

Figure S1. The schematic diagram of electron configurations for (a) LS Co^{3+}, (b) IS Co^{3+}, and (c) HS Co^{3+} in $LaCoO_3$.

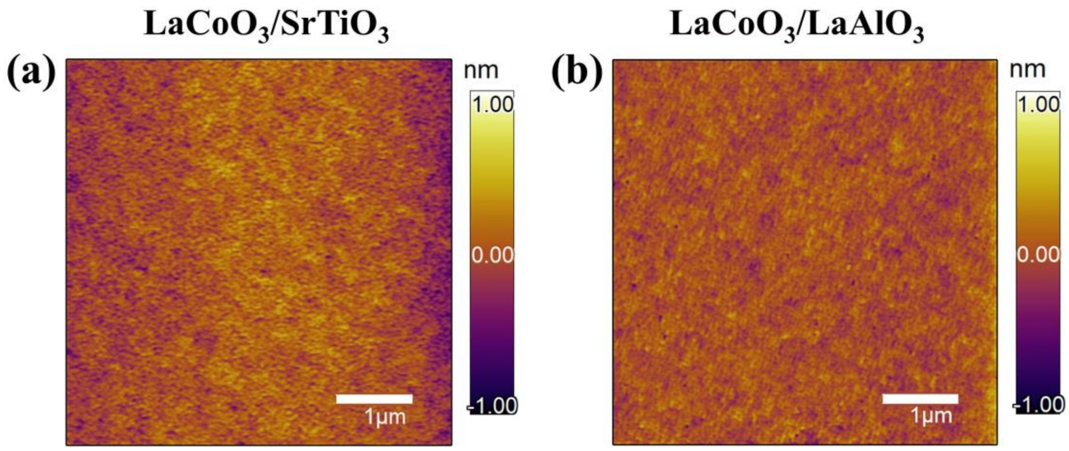

Figure S2. The surface topographic image of (a) $LaCoO_3/SrTiO_3$ and (b) $LaCoO_3/LaAlO_3$ films, respectively. The root-mean-square (RMS) roughness for $LaCoO_3/SrTiO_3$ and $LaCoO_3/LaAlO_3$ films is 170 pm and 120 pm, respectively.

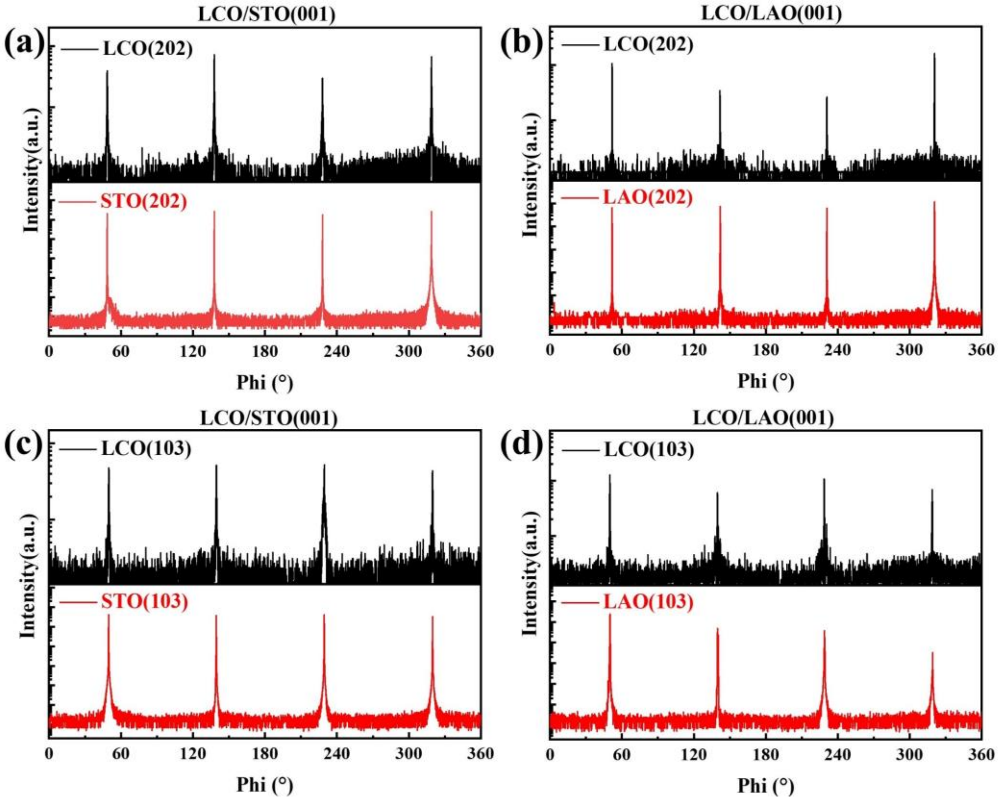

Figure S3. XRD phi scans for LCO/STO and LCO/LAO.

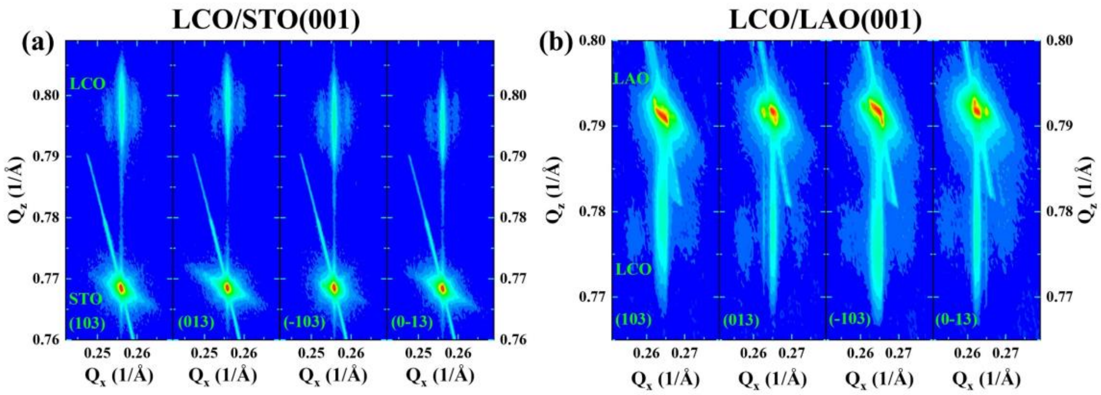

Figure S4. Reciprocal space mappings (RSM) of LCO films grown on a) STO and b) LAO around the substrate's (103), (013), (-103) and (0-13) reflections, respectively.

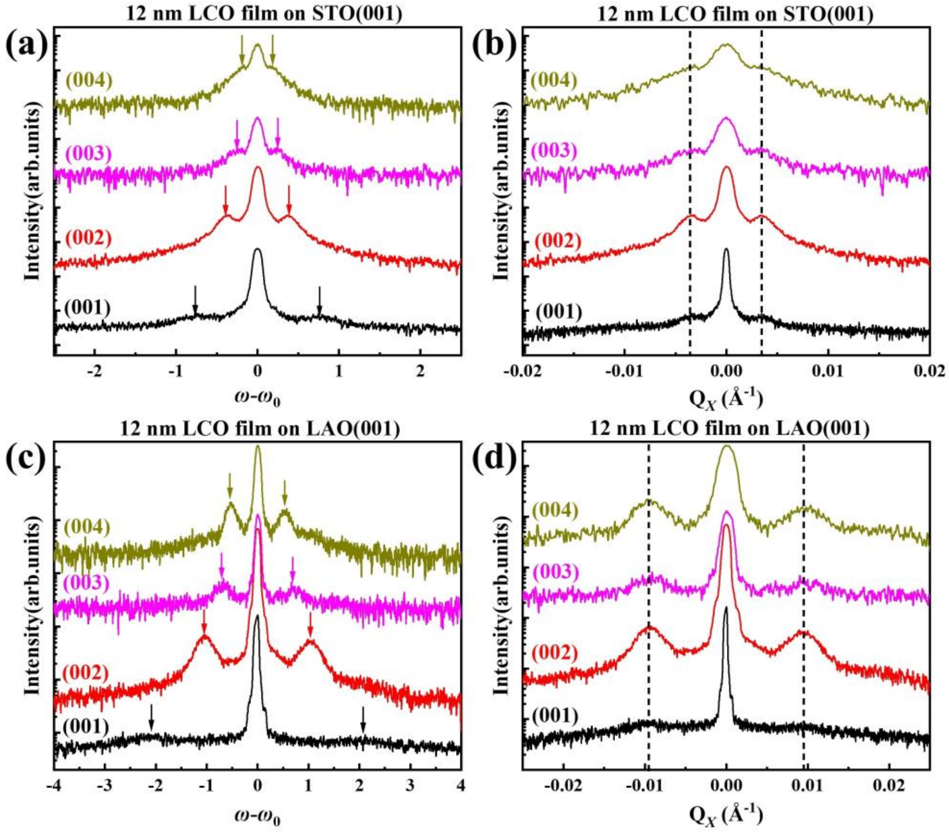

Figure S5. (a, c) Rocking curves around LCO 00*l* (*l* = 1, 2, 3, and 4) reflections for LCO/STO(001) and LCO/LAO(001), respectively. (b, d) Transformed rocking curves in reciprocal space lattice. for LCO/STO(001) and LCO/LAO(001), respectively.

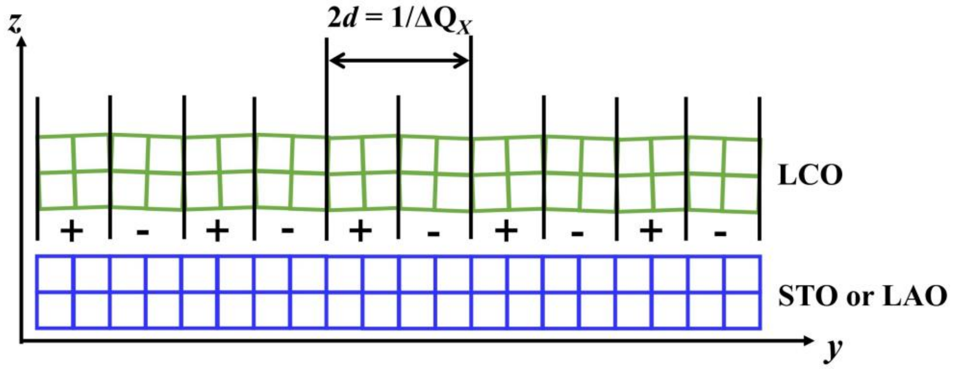

Figure S6. Real space sketch to the LCO film structure grown in average pseudomorphically on top of a rigid, STO or LAO substrates.

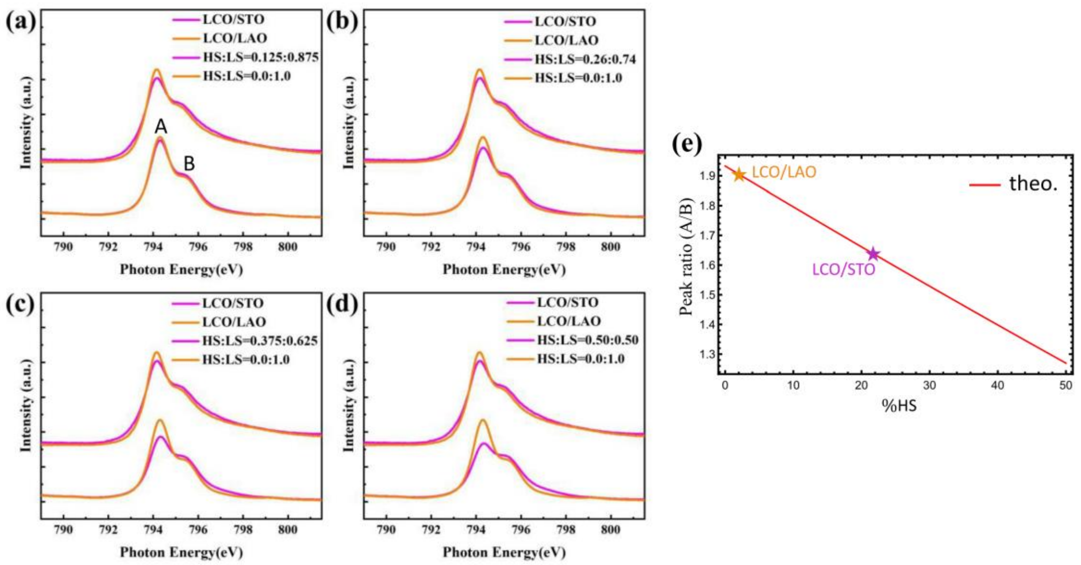

Figure S7. (a-d) The experimental Co L_2 XAS spectra of LCO/LAO and LCO/STO at 30 K and the corresponding theoretical isotropic spectra in the LS-HS scenario with different HS/LS ratio. (e) Comparison of the intensity ratio of peak A to peak B as indicated in Figure S7a taken from the calculated XAS (red line) and experimental XAS (stars). The error bar of determining the spin state configuration by XAS is estimated around 3%.

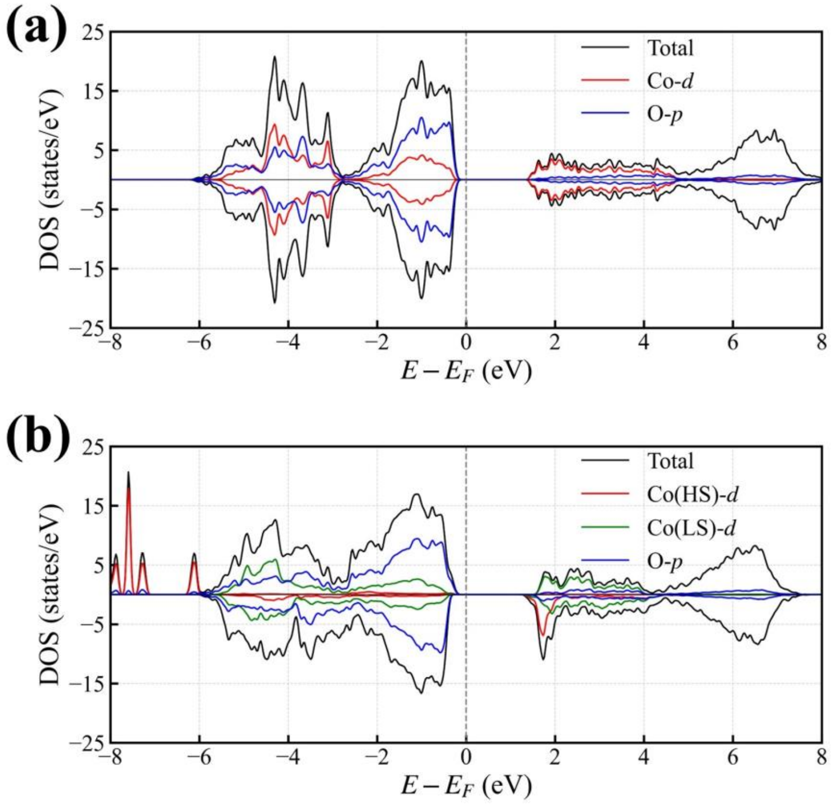

Figure S8. The total and atomic projected densities of states (DOS) for $LaCoO_3$ thin films under different substrates. (a) The low-spin state of $LaCoO_3$ thin films under $LaAlO_3$ substrate. The black, red and blue curves are total, Co-*d* and O-*p* projected DOS, respectively. (b) The mixed-spin state of $LaCoO_3$ thin films under $SrTiO_3$ substrate. The black, red, green and blue curves are total, high-spin (HS) Co-*d*, low-spin (LS) Co-*d* and the O-*p* projected DOS, respectively. In both panels, E_F is the Fermi level.

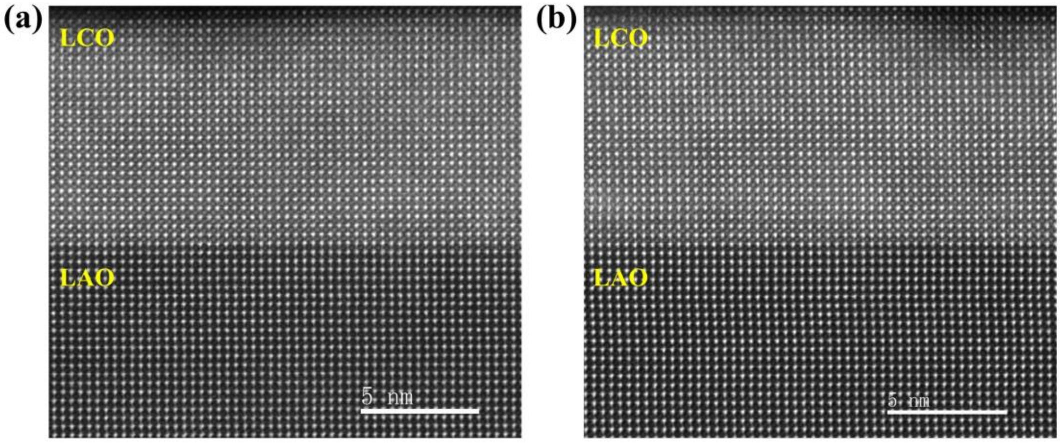

Figure S9. High-resolution HAADF STEM image of a LCO film grown on a LAO substrate with different electron dose condition; (a) low- and (b) high-dose conditions.

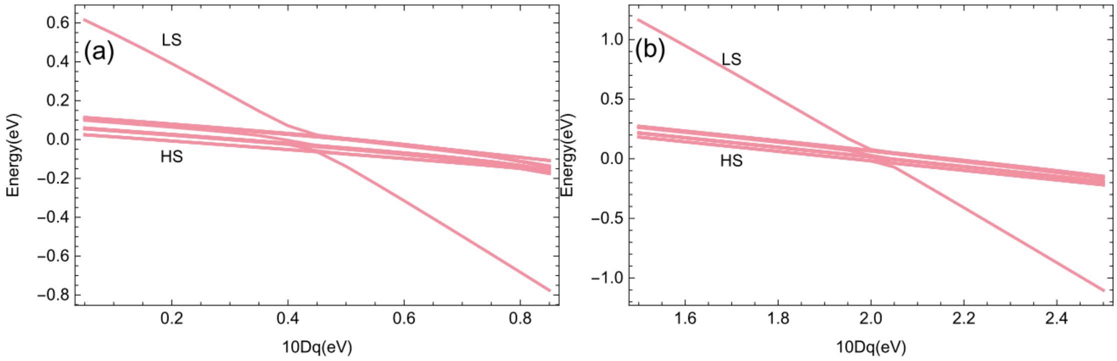

Figure S10. The energy level diagram as a function of 10Dq calculated under the three configurations model (a) and the single configurations model (b).

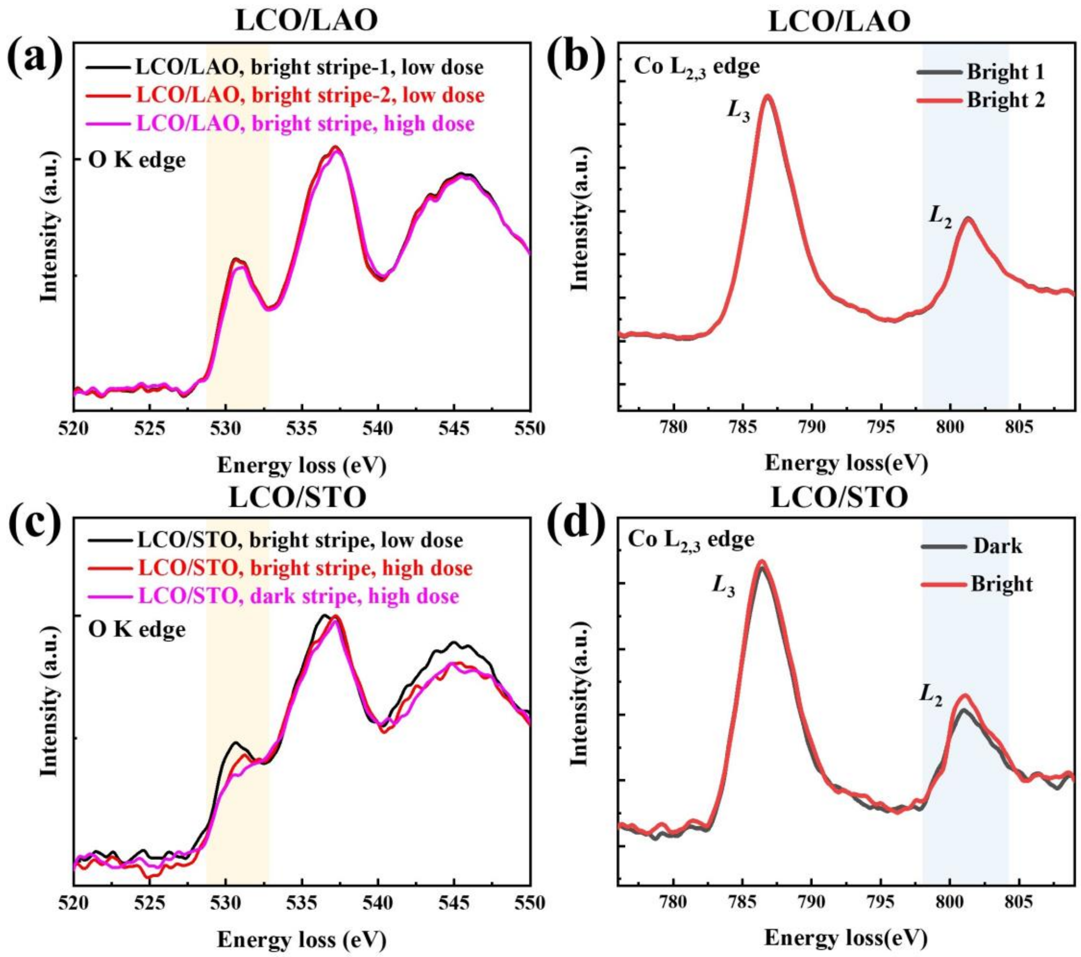

Figure S11. (a, c) O-*K* edge and (b, d) Co-*L* edge EELS spectra from line scans taken along the bright and dark contrast planes for LCO/LAO and LCO/STO under low and high dose electron beam conditions. It can be seen that there is a considerable difference in the peak around 530 eV between LCO/LAO and LCO/STO, which is due to the fact that LCO/STO is more prone to electron beam damage. The spectra around 530 eV are usually attributed to the O 2*p* hybridized with Co 3*d*. The presence of the oxygen vacancies caused by the electron beam irradiation damage, which form the Co^{2+} impurities, will reduce the peak intensity around 530 eV because the highly ionic nature of the Co^{2+} impurities forms the weaker hybridization with O 2*p* states. In Figure S11(a) we show the O K edge EELS spectra of LCO/LAO. It can be seen that the spectra got only a small drop in intensity around the 530 eV peak by taking with higher dose of electron beam irradiation, indicating that the compressive LCO film is less prone to the electron beam irradiation damage, i.e. the formation of oxygen vacancies. In contrast, as shown in Figure S11(b), the intensity around the 530 eV peak of LCO/STO decreases

more when the higher dose electron beam is used to take the EELS data. It is noted that the EELS spectrum taken in the dark stripe has a lower intensity of the 530 eV pre-peak than that recorded in the bright stripe, indicating more oxygen vacancies in the dark stripe. The spectra around 530 eV are usually assigned to the O 2*p* band hybridized with Co 3*d* state.[32] The presence of the oxygen vacancies caused by the electron beam irradiation damage, which form the Co^{2+} impurities, will reduce the EELS peak intensity around 530 eV because the highly ionic nature of the Co^{2+} impurities forms the weaker hybridization with O 2*p* states. These findings indicate that the oxidation state of Co along the dark stripes has been reduced more by a relatively high dose of electron beam. Previously studied has suggested that the HS state is more prone to the oxygen vacancies than the LS state,[38-39] indicating that the location of the dark stripes is where HS Co^{3+} is present.
